# Cloning, heterologous expression, and characterization of a novel thioesterase from natural sample

**DOI:** 10.1016/j.heliyon.2021.e06542

**Published:** 2021-03-24

**Authors:** Suharti, Gita Mahardika, Raissa, Laksmi Dewi, Heni Yohandini, Made Puspasari Widhiastuty, Raden Aditya Wibawa Sakti, Setyanto Tri Wahyudi, Akhmaloka

**Affiliations:** a Biochemistry Research Group, Faculty of Mathematics and Natural Sciences, Institut Teknologi Bandung, Jl. Ganesha 10, Bandung, Indonesia; b Department of Chemistry, Faculty of Science and Computer, Universitas Pertamina, Jl. Teuku Nyak Arief, Simprug Kebayoran, South Jakarta, Indonesia; c Department of Chemical Engineering, Faculty of Industrial Engineering, Universitas Pertamina, Jl. Teuku Nyak Arief, Simprug Kebayoran, South Jakarta, Indonesia; d Department of Chemistry, Faculty of Mathematics and Natural Sciences, Universitas Sriwijaya, Jl. Raya Palembang Prabumulih KM 32, Indralaya-Ogan Ilir, Indonesia; e Department of Physics, IPB University, Bogor, 16680, Indonesia

**Keywords:** Thioesterase, Cloning, Expression, Extreme thermostable, Natural sample

## Abstract

A novel thioesterse gene was successfully cloned and sequenced directly from natural sample of Domas Hot Spring, West Java, Indonesia. Homological analysis of the sequence showed that the gene appeared high homology to thioesterase genes with the highest to a putative thioesterase gene from uncultured *Acidilobus sp. JCHS* at 66% identity. However, phylogenetic analysis showed that the protein was separated from the branch with other known thioesterases. The size of the gene is around 500 base pairs, lied into 2 kb DNA fragment from a random PCR amplicon. The gene was overexpressed in *Escherichia coli*, a dominant band appeared at 17 kDa in SDS-PAGE with expression level at around 32% of total proteins. The activity of the purified protein using acetyl-CoA as substrate showed that the protein exhibited thioesterase activity. Furthermore, the enzyme also showed esterase activity on p-nitrophenyl ester as substrate. Detail characterization of esterolytic activity showed that the enzyme preferred p-nitrophenyl decanoate as substrate. The optimum activity of the enzyme was at 80 °C and pH 8. Activity of the enzyme was maintained after incubation at 80 °C up to 24 h. In addition, the enzyme was favorable on polar organic solvents. All the data obtained suggested that the enzyme is a novel alkaline thermostable thioesterase.

## 1 Introduction

Carboxylic esterase is family of enzymes catalyzing hydrolysis, synthesis or transesterification of ester bonds reactions. In aqueous conditions, the enzyme catalyzes the hydrolysis of ester bonds to produce alcohol and carboxylic acids. The enzymes also play a role in the reverse reaction or transesterification reaction in organic solvents [1]. Ester carboxylic hydrolases are classified in two major groups, lipases and esterases. The significant differences of these two hydrolases are indicated by the length of substrate chain. Esterases play a role in the hydrolysis of short-chain acyl (less than 10 carbon atoms) and do not work on the substrate that forms micelles [2]. Other groups include in esterases are, aril esterase and phospholipase.

Most ester hydrolase are grouped in α/β-hydrolase family. Ester hydrolase also has structural and functional characteristics, including α/β-hydrolase folds, catalytic triads and independent activity of co-factors [3]. The catalytic triad region, which is a sustainable region, usually consists of nucleophilic serine in the GXSXG penta peptide motif, where X is any residue, or acid, aspartic or glutamate, residues that have hydrogen bonds to histidine residues [3, 4].

Thioesterase (EC 3.1.2.-) is a member of carboxylic esterases, most of them belong to α/β hydrolase families usually found in the C-terminal as a much larger of non-ribosomal peptide syntase, NRPS domain or type I fatty acid synthases, FAS or polyketide synthases, PKS enzymes [5]. Thioesterase is a family of hydrolases α/β containing a classic catalytic triad of nucleophile-histidine-aspartate and often do macrocyclizations instead or in addition to hydrolysis [6]. In contrast, the majority of thioesterase hotdog are different since they are found to be free (Type II) thioesterase. Some thioesterase hotdogs do not use classical catalytic triad or histidine from the dehydrolase/hydrolase hotdog group. The effect of the helical polarization of a hotdog may be the only active site feature of the dehydrolase/hydrolase group that brings to thioesterases hotdogs. Many thioesterase hotdogs found containing glycine residues which are conserved at the αHD end, and the backbone of this amide residue, is equally involved in most of the proposed mechanisms that function as part of the oxyanion hole [7]. The activities of some thioesterases were overlap with the esterase, since both enzymes belong to a member of ester hydrolase family [4] some thioesterases exhibit esterase activity [8, 9] and vice versa [10, 11].

Carboxylic esterase could be found in all three domains of life (bacteria, eukaryote, and archaea). The enzyme is one of the most exploited biocatalyst groups and shows an important role [12, 13] in many industries. Various industries such as detergents, food, biodiesel, organic synthesis and medical biotechnology, use esterase in the process. However, using enzymes in many industrial processes have many limitations. Parameters such as high stability in organic solvents, wide substrate specificity [14] and stereo selectivity [15] are needed to make the enzymes attractive for biocatalysts. Some industries operate at high temperature and use organic solvent. Esterases from hyperthermophiles often show high intrinsic stability, making it attractive to use for various biotechnological applications.

Some thermostable lipases and esterases from thermophilic organisms were cloned and characterized, from various natural sources such as hot springs, craters or compost, both through cultivation [14, 16, 17, 18, 19, 20, 21, 22] and metagenomics approaches [23]. In this report, cloning, heterologous expression, and characterization of a novel gene from natural sample of Domas Hot spring, West Java, namely *TESITB* was described.

## 2 Material and methods

### 2.1 DNA isolation

Total community DNA were isolated directly from Domas Hot Spring, Tangkuban Perahu, West Java, Indonesia, as described by previous report [24]. Pellet cells were mixed with 600 μL DNA extraction buffer (100 mM tris-Cl, 100 mM EDTA, 100 mM sodium phosphate, 1.5M NaCl, 1% CTAB, pH 8). The mixture was then added 30 μL proteinase K 10 mg/mL and 1 g of sterile sea sand in 2 mL microtube and then shaken at 37 °C 150 rpm for 30 min. 60 μL of SDS 10% was added and then incubated at 60 °C for 2 h with gentle shaking. The mixture was then precipitated by centrifugation at 6000 g for 10 min. The supernatant was collected in new sterile 1.5 mL tube, and then added by 0.6 volume of isopropanol, gentle shaking and incubated at room temperature for an hour. The pellet of crude nucleic acid was obtained by centrifugation at 12,000 g for 30 min at room temperature. The pellet was then washed by 70% of cold ethanol and resuspended on sterile ddH_2_O to give a final volume of 50 μL DNA solution.

### 2.2 PCR and cloning

Preparation of primers, PCR technique, and cloning were based on previous report [25]. A pair of primers (FE: 5^’^- GTA GCC ATA GAC GTG AGG T-3^’^; RE: 5′- GCC TCT CTA AGT CTT GAA GAA CG-3^’^) were used to amplify the gene randomly from total community DNA sample. PCR reaction was performed by using Sso fast supermix (BioRad). 20 μL of PCR reaction (10 μL of Sso fast, 2 pmoL of primer pair, 1 μL of community DNA amplification). PCR was carried out into five steps. An initial denaturation step was carried out at 98 °C for 3 min. The PCR was carried out for 31 cycles, each cycle was used denaturation at 98 °C, 30 s; annealing with gradient temperature from 56 to 46 °C for 30 s; elongation at 72 °C for 3 min; extension reaction at 72 °C for 10 min and cooling at 12 °C for 10 min.

### 2.3 Homological analysis

Homological analysis was performed by aligning the DNA sequence with NCBI data using BLASTN program (https://www.ncbi.nlm.nih.gov/) [26]. Prior the homological analysis, the sequence was exposed to ORF finder for determining the coding region. The coding region was translated *in silico* based on BLASTP program. Alignment of amino acid sequence was performed by Clustal X program and visualized using genedoc program. Phylogenetic tree was constructed based on maximum likehood method using Jones-Taylor-Thomson (JTT) model with MegaX (https://www.megasoftware.net/) [27].

### 2.4 Structure modeling and molecular dynamic simulation

Three-dimensional structure modeling was performed by superposition of amino acid sequence with protein structures in Protein Data Bank, PDB (https://www.rcsb.org/). The amino acid sequence of the sample was submitted to Swiss-Model server (https://swissmodel.expasy.org/) [28]. Visualizing of 3-dimensional models were used PyMol viewer program (https://pymol.org/2/). All simulation were carried out in the AMBER 18 program package (https://ambermd.org/) [29] at 353 K. The simulation began with the minimization, heating, equilibration, and production run at the classical mechanics level.

### 2.5 Sub-cloning and heterologous expression

Thioesterase gene was isolated and sequenced from recombinant plasmid containing 2 kb of DNA fragment. A pair of primers was synthesized based on nucleotide sequence of the gene to amplify the whole gene and inserted to expression vector. The sequence of forward primer is FTEex 5′-GCAGTATTG**CATATG**ATGTCGTTG -3′ containing a unique *Nde*I site and the reverse primer sequence is RTEex 5′- GGTCATGCC**CTCGAG**GCTTCCTGA -3′ containing a unique *Xho*I site. PCR was started with denaturation at 95 °C for 4 min followed by reaction cycles. Each reaction cycle was accomplished at 95 °C for 30 sec, 30 sec for annealing at 46 °C and 72 °C for elongation, 45 sec. After the reaction cycles, the elongation process was continued at 72 °C for 10 min and cooling at 12 °C for 10 min.

The amplicon containing the whole gene was ligated to pJET 1.2/blunt using CloneJET PCR Cloning Kit (Thermo Fisher Scientific). The product was transformed into *E. coli* TOP 10. The plasmid was isolated and digested with *Nde*I and *Xho*I. Subsequently, the thioesterase gene then ligated into the expression vector pET30a (Novagen, USA).

The recombinant expression vector containing thioesterase gene was then transformed into *E. coli* BL2 (DE3) and overexpression of TesITB gene was induced by IPTG (1 mM) at the mid exponential growth (OD_600_ = 0.6), followed by 4 h incubation at 37 °C. The cells were harvested by centrifugation at 6000 g, 4 °C for 30 min and resuspended in 10 mM Trish-HCL buffer pH = 7. The cells were disrupted by sonication and the crude protein sample was treated at 60 °C for 30 min after centrifugation at 16,000 g, 4 °C for 15 min. The resulting supernatant was then applied to an once step purification through Ion Metal Affinity Chromatography through NiNTA resin (BioRad). The column (0.8 × 4 cm) was washed and bound protein were eluted by Tris-HCL buffer pH = 7 containing 250 mM NaCl and imidazole gradient (20–250 mM). Protein concentration was determined with bovine serum albumin (BSA) as standard. Sodium dodecyl sulfate-polyacrylamide gel electrophoresis (SDS-PAGE) was performed with 12% polyacrylamide gel, as described by Smith [30].

### 2.6 Thioesterase activity assay

Thioesterase activity was determined by colorimetric method using acetyl-CoA as substrate [31]. The activity was measured by coupling thioester hydrolysis with fast reaction of 5,5′-dithiobis (2-nitrobenzoic acid) with the free thiol (CoA) resulting from the hydrolysis of acetyl-CoA substrates [32].

**Figure:**
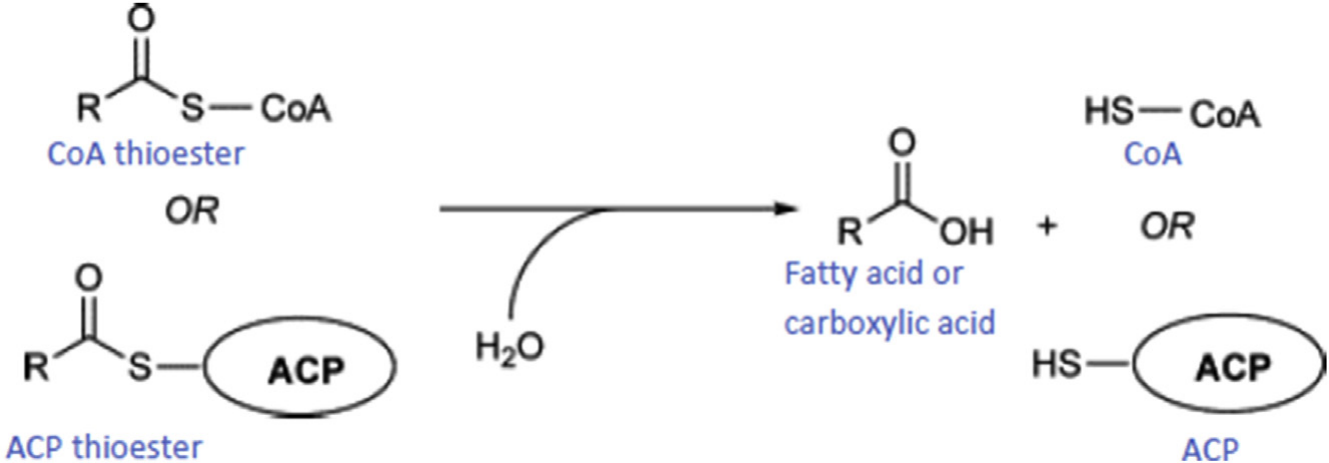

A total enzyme concentration of 10 mM was pre-incubated for 1 h with 1 mM 5,5′-dithiobis (2-nitrobenzoic acid) in 50 mM K^+^HEPES (pH 8.0) at 25 °C followed by addition of acety-CoA (1 mM) in a total volume of 200 μL. The mixture was further incubated at 25 °C for period of time. The reaction was stopped by incubating the mixture on ice. The product was monitored by spectrophotometer at 412 mM. one unit was defined as amount of enzyme to release 1 μmol product per min and the extinction coefficient at 162 cm^2^ mol^−1^.

### 2.7 Esterase activity assay

Esterase activity was determined by modified colorimetric method [21] using *p*-nitrophenyl fatty acid ester as substrate. 300 μL protein solution was added by 900 μL substrate mixture (sodium phosphate buffer 0.05M pH 8: ethanol: substrate solution 10 mM = 95:4:1). Reaction mixture was incubated at 50 °C pH 8 for 15 min. The reaction was stopped by incubated the mixture on ice.

### 2.8 Substrate specificity

Substrate preference of the enzyme was determined by using p-nitrophenyl acetate (pNPA), p-nitrophenyl butyrate (pNPB), p-nitrophenyldecanoate (pNPD), p-nitrophenyl laurate (pNPL), p-nitrophenylmiristate (pNPM) and p-nitrophenyl palmitate (pNPP) as substrates. Reaction mixture was incubated in standard reaction as described before.

### 2.9 Optimum temperature

Optimum temperature assay was performed using pNPD as substrate with temperature range at 30–90 °C, pH 8. Assayed was performed in standard reaction as described before.

### 2.10 Optimum pH

Optimum pH assay was performed using pNPD as substrate and reaction mixture was incubated at various pH (sodium phosphate buffer 0.05 M for pH 6–8 and glycine-NaOH buffer for pH 9–10) for 15 min.

### 2.11 Enzyme assay in organic solvents

Methanol, ethanol, isopropanol, acetone, acetonitrile, chloroform and n-hexane at 3% concentration were used to probe the preference of the enzyme in the present of organic solvent. The assay was performed at 80 °C, pH 8 and other parameters used as standard reaction described before.

### 2.12 Thermostability assay

To probe thermostability at 80 °C, the enzyme was incubated at 80 °C for 2, 4, 6, 8, 18 and 24 h. 300 μL of protein solution was added by 900 μL of substrate mixture (sodium phosphate buffer 0.05 M pH = 8: ethanol: substrate solution 10 mM = 95: 4: 1). Reaction mixture was incubated at 80 °C, pH 8 for 15 min.

## 3 Results and discussions

### 3.1 Sequence and homology of TesITB

**Figure 1:**
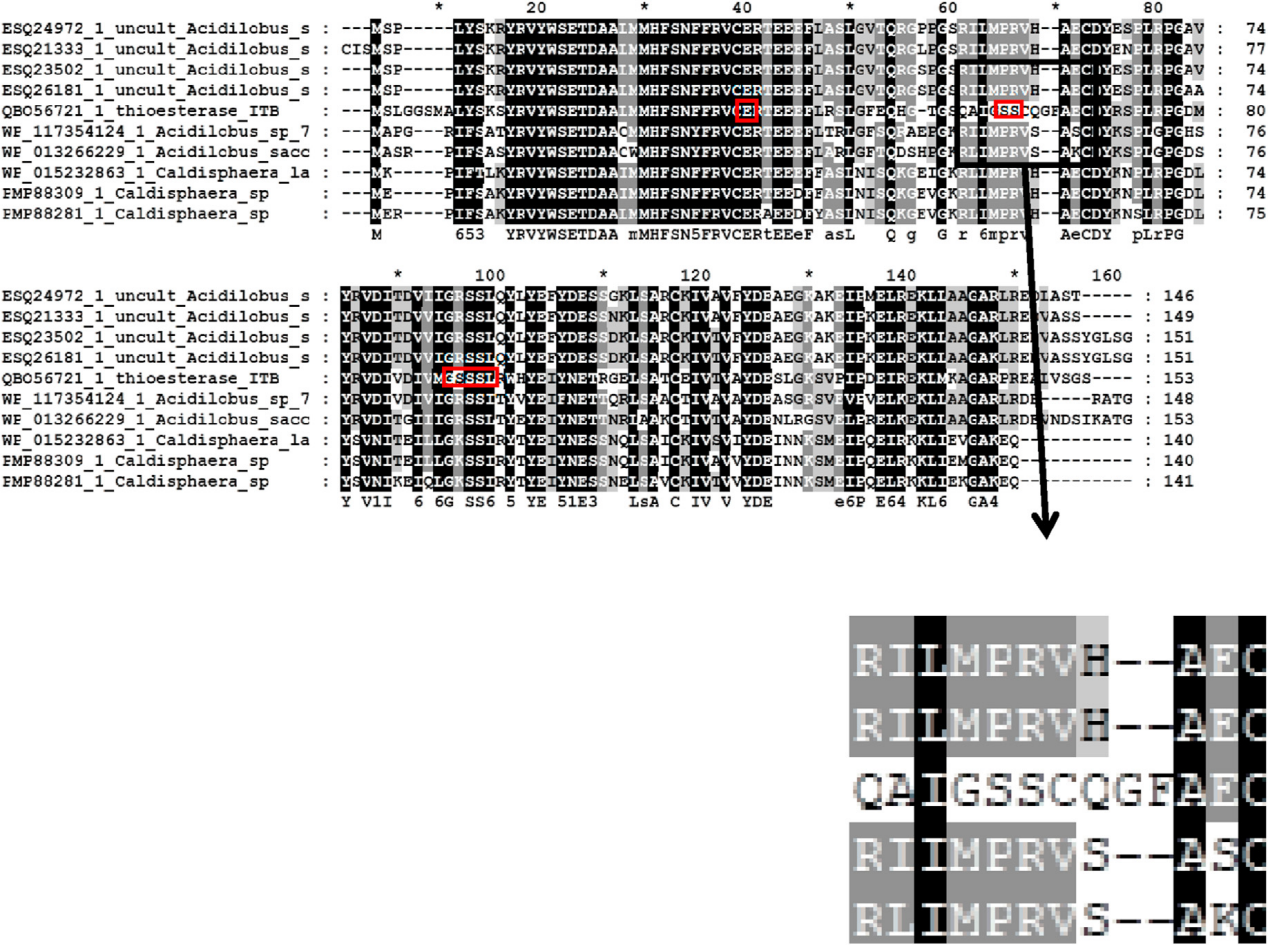
Amino acid sequence alignment of TesITB with 9 best homologs. The black colours were conserved region of thioesterase. Significant differences show inside box (amino acid residues from 60-70). Sequence region of RI/LIMPRU was replaced by QAIGSSC and an insertion of GF residues in TesITB. The red rectangles are shown the catalytic triad. 1st red rectangle is E37, 2nd red rectangle is 61SS62, and 3rd red rectangle is 91GSSSL95.

**Figure 2:**
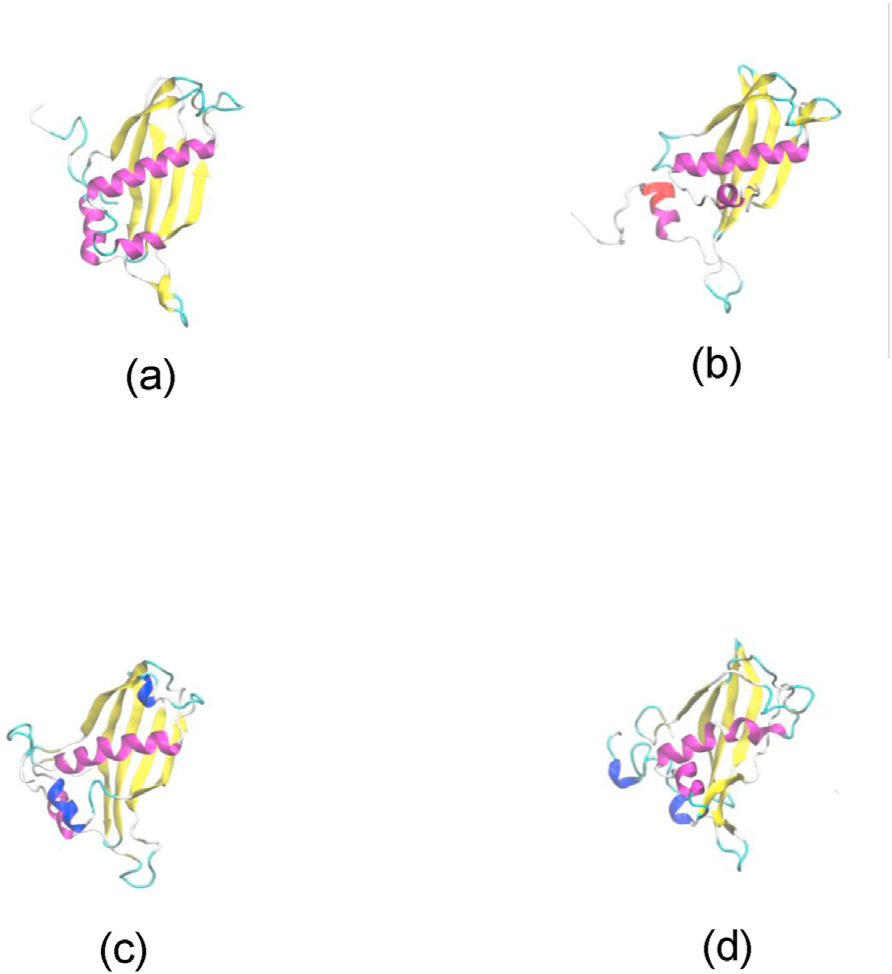
Structure of TesITB in (a). water, (b) ethanol, (c). acetonitrile, and (d). n hexane. The structure was taken after 50 ns production runs using Swiss Model.

**Figure 3:**
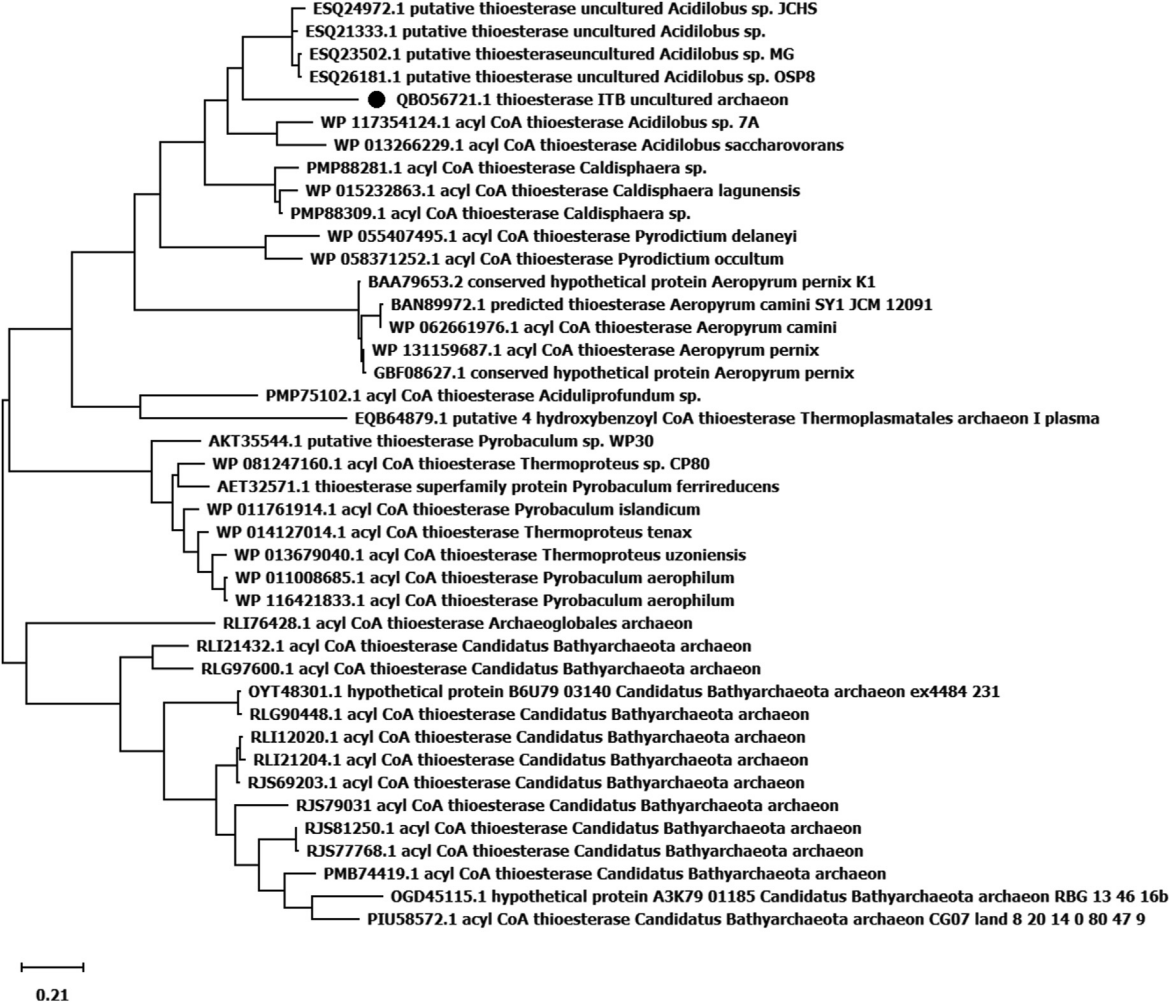
Phylogenetic tree of TesITB maximum likelihood and JTT matrix – based mode (MEGA-X) were used to construct the tree based on the comparison among the amino acid sequence of TesITB and of the 40 best homolog sequence from the GenBank.

2kb DNA fragment containing thioesterase gene was amplified from chromosomal DNA directly isolated from water hot spring by using random primers [25] and sequenced. The nucleotide sequences of 2 kb DNA was deposited in the gene bank with accession number of MT 993376. An ORF of gene was detected with size at around 500 bp. Further analysis on the sequence appeared that the gene showed high homology to family of thioesterase with the highest homology to putative thioesterase from *Acidolabus sp*, at 66% identities (Table 1). Homological analysis showed that the protein contains similar conserved region with other known thioesterase [33]. The region shows a hotdog motif belonged to thioesterase and thiohidrolase [34]. High homology of the region was also appeared with 4-hydroxy benzoyl-CoA thioesterase (4-HBT) known as catalyse for degradation of 4-chloro benzoate to 4-hydroxy benazoat [35]. The protein showed a catalytic triad ... E...S...S.. (Figure 1). Comparison of the protein to 9 other best homolog thioesterase appeared that TesITB contains significant different sequence at residues 60–70 (Figure 1). Thioesterase ITB contains QAI GSSC motif while the others are RI/LI MPRV. In addition, there are two residue of amino acids (GF) inserted to the protein. Structure prediction of TesITB using Swiss Model showed that there is hotdog motif (Figure 2) as other known thioesterase [34]. Further analysis on variation of sovents, the structure of TesITB was seemed more compact by decreasing polarity of the solvent (Figure 2). Phylogenetic analysis with 40 other best homologs showed that TesITB forms same group with *Acidilobus* thioesterase (Figure 3), however, detail analysis seems that the protein was out of branch. From homological and computer modelling analysis suggested that TesITB is thioesterase however based on phylogenetic analysis the enzyme is unlikely same as other known thioesterases. The nucleotide sequence of the gene, namely Thioesterase (*TES*) ITB, was deposit in the GeneBank with accession number of QB 056721.1.

**Table 1:** Homological analysis of TesITB. The protein shows high homology with other Thioesterase, the highest identity was shown with putative Thioesterase from uncultured Acidilobus sp.

Accesion number	Description	Identity (%)
ESQ24972.1	putative thioesterase [uncultured *Acidilobus* sp. JCHS]	66
ESQ23502.1	putative thioesterase [uncultured *Acidilobus* sp. MG]	65
ESQ26181.1	putative thioesterase [uncultured *Acidilobus* sp. OSP8]	65
ESQ21333.1	putative thioesterase [uncultured *Acidilobus* sp. CIS]	64
WP_117354124.1	acyl-CoA thioesterase [*Acidilobus* sp. 7A]	61
WP_015232863.1	acyl-CoA thioesterase [*Caldisphaera lagunensis*]	61
PMP88281.1	acyl-CoA thioesterase [*Caldisphaera* sp.]	59
WP_013266229.1	acyl-CoA thioesterase [*Acidilobus saccharovorans*]	58
PMP88309.1	acyl-CoA thioesterase [*Caldisphaera* sp.]	58
WP_055407495.1	acyl-CoA thioesterase [*Pyrodictium delaneyi*]	51

### 3.2 Heterologous expression of thioesterase ITB

**Figure 4:**
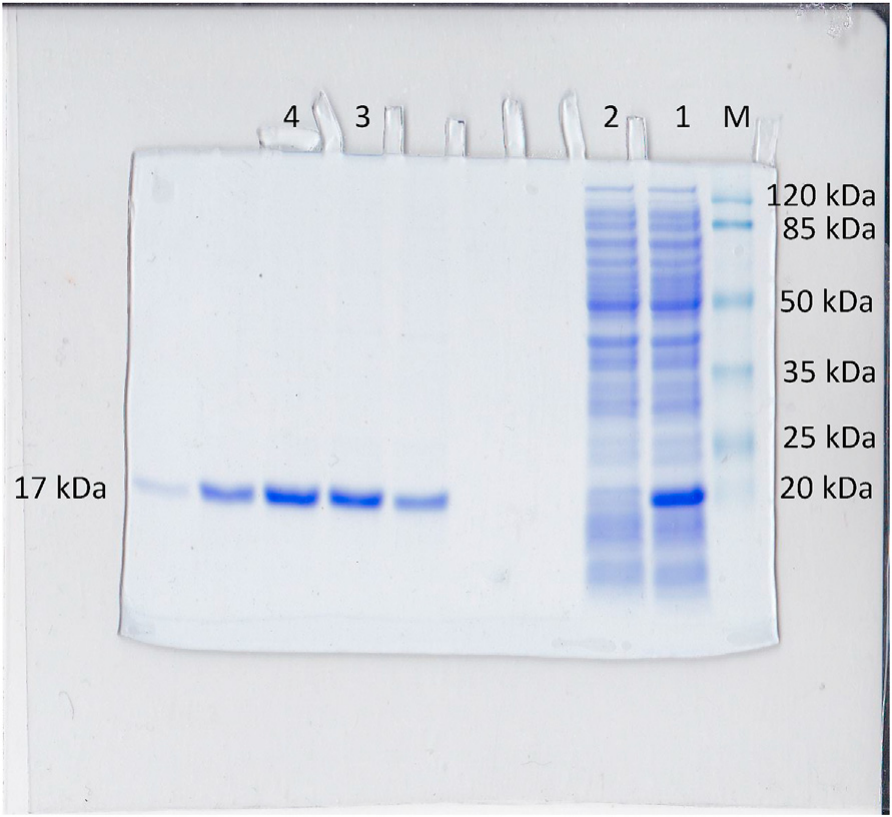
Electrophoregram of SDA-PAGE using 12% acrylamid. Marker protein (M); crude extract of *E. coli* containing overexpression of TesITB (1); Thio through of crude extract after binding to NiNTA residue (2); Purified TesITB through IMAC NiNTA purification.

**Figure 5:**
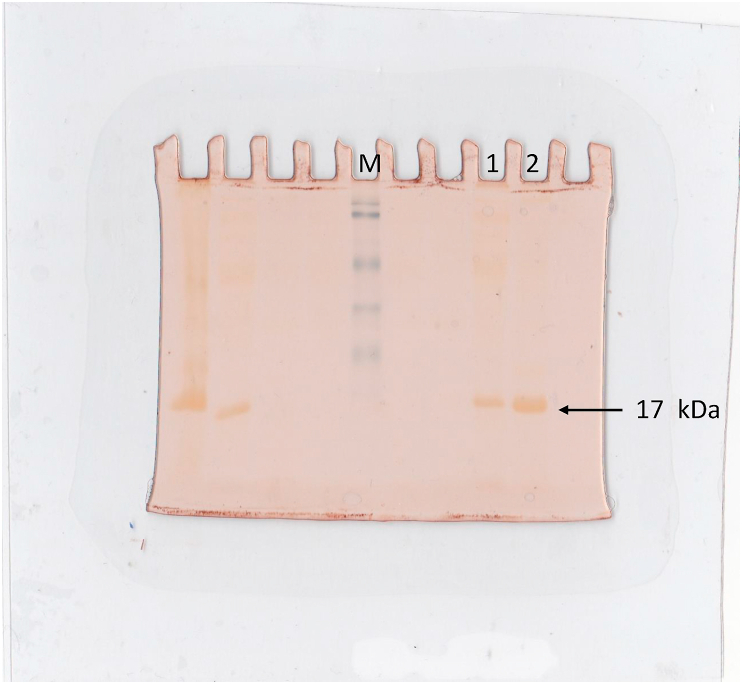
Zymografic profile of TesITB. Zymography assay was carried out using 1- naftyl acetate as substrate. A clear band of positive result with the size around (1,2); 25KD of marker protein (M).

*TES* gene was isolated from recombinant plasmid containing 2 kb inserted fragment using PCR and successfully overexpressed in *E. coli* BL21 (DE3) by inducing with IPTG. Crude extract was isolated and visualized using SDS-PAGE (Figure 4, lane 1). A dominant band with size at around 17 kDa appeared as result of high expression of the gene. Densitometric analysis of the crude extract revealed that the protein was expressed at around 32% of total proteins. The crude extract of protein was assayed to probe the esterase activity using 1-naphthyl acetate. Zymografic analysis showed a clear single band revealed with the size of 17 kDa as expected (Figure 5). The enzyme was successfully purified by IMAC (NiNTA) and confirmed a single band on SDS-PAGE (Figure 4, lane 3 and 4).

### 3.3 Activities of purified TesITB

**Figure 6:**
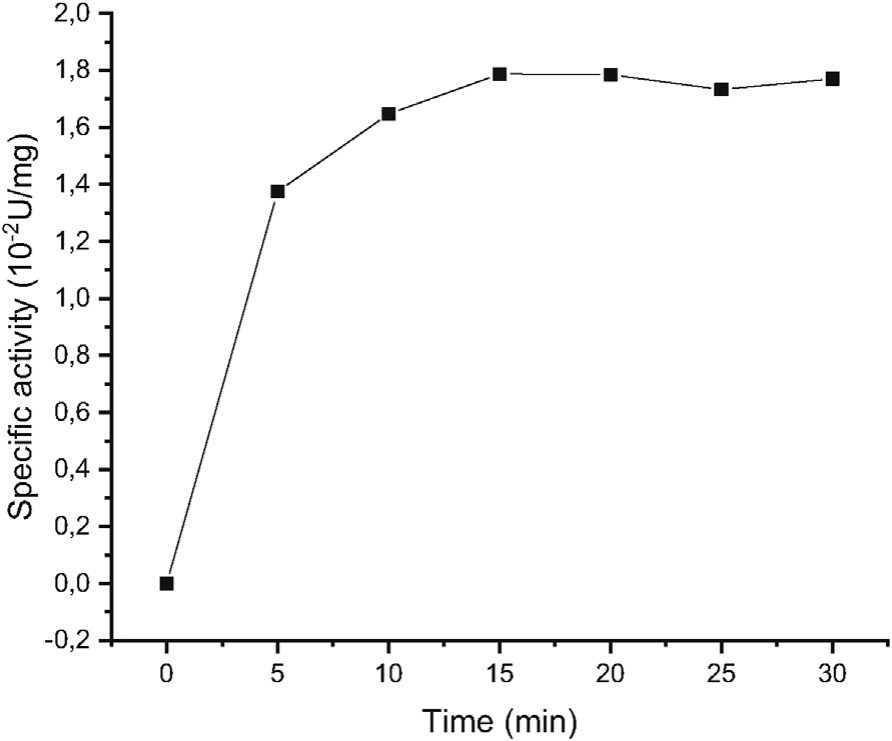
Thioesterase activity of the enzyme using Acetyl-CoA as substrate. The assay was carried out at 25 °C.

Following the confirmation of the esterase activity on the crude extract (Figure 5), two assays were performed to probe thioesterase and esterase activities of the purified TesITB. Thioesterase activity was determined using standard method and acetyl-CoA as substrate [36]. The result showed that TesITB exhibited thioesterase activity (Figure 6). Since acetyl-CoA is relatively unstable substrate at high temperature and alkaline pH, further characterization of the enzyme was used para-nitrophenyl acyl ester (p-NP-acyl ester) as substrate.

**Figure 7:**
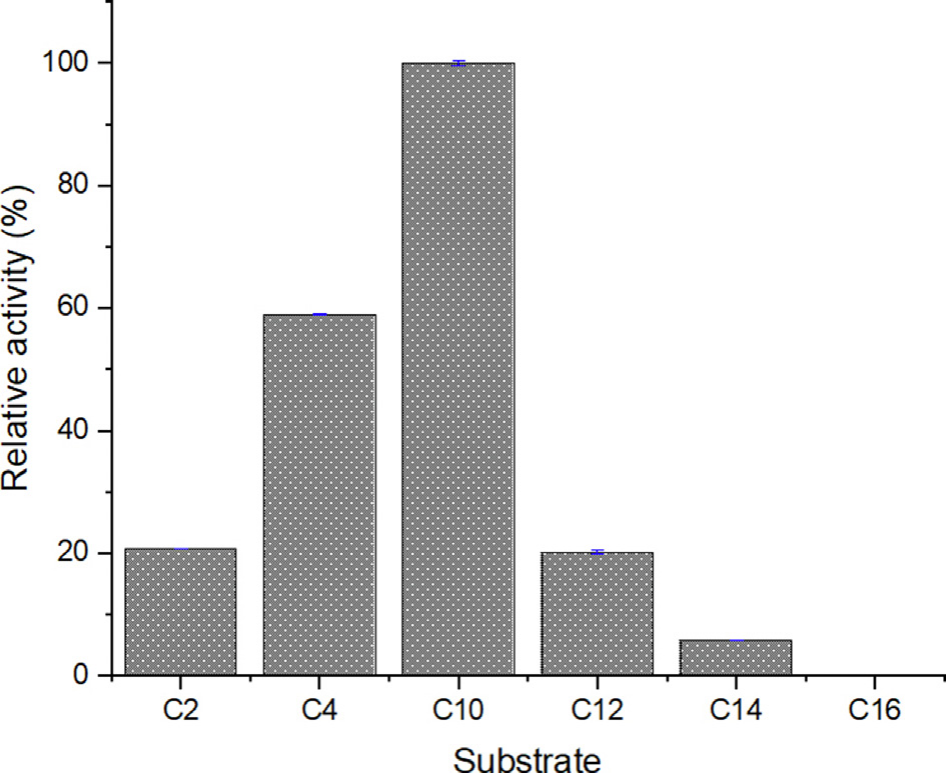
Relative esterase activity of the enzyme on variation of substrates. The assay was carried out at 50 °C, pH 8.

Esterase activity of TesITB was performed using para-nitrophenyl acyl ester as substrate as described on previous report [21]. Highest activity of the enzyme was shown using para-nitrophenyl decanoate (Figure 7). Most of thioesterases/esterases are specific to a particular type of substrate especially on carbon length of substrate [9]. Geometry and hydrophobicity of catalytic pocket determine chain length specificity of esterase [22, 37].

**Figure 8:**
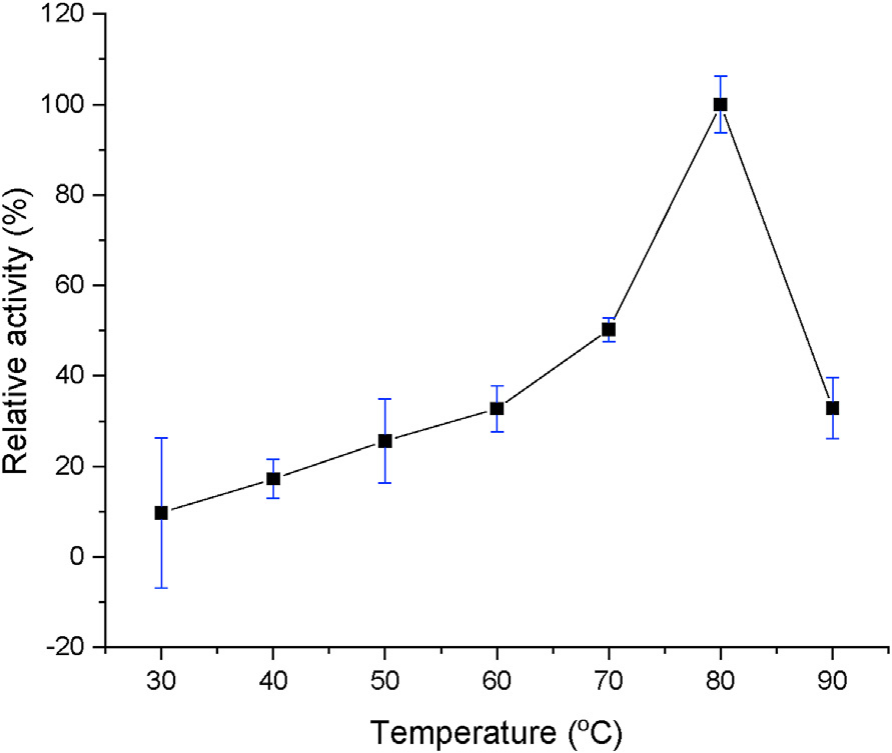
Relative esterase activity of the enzyme in range of temperature. The assay was performed at pH 8 using pNP-decanoat as substrate.

**Figure 9:**
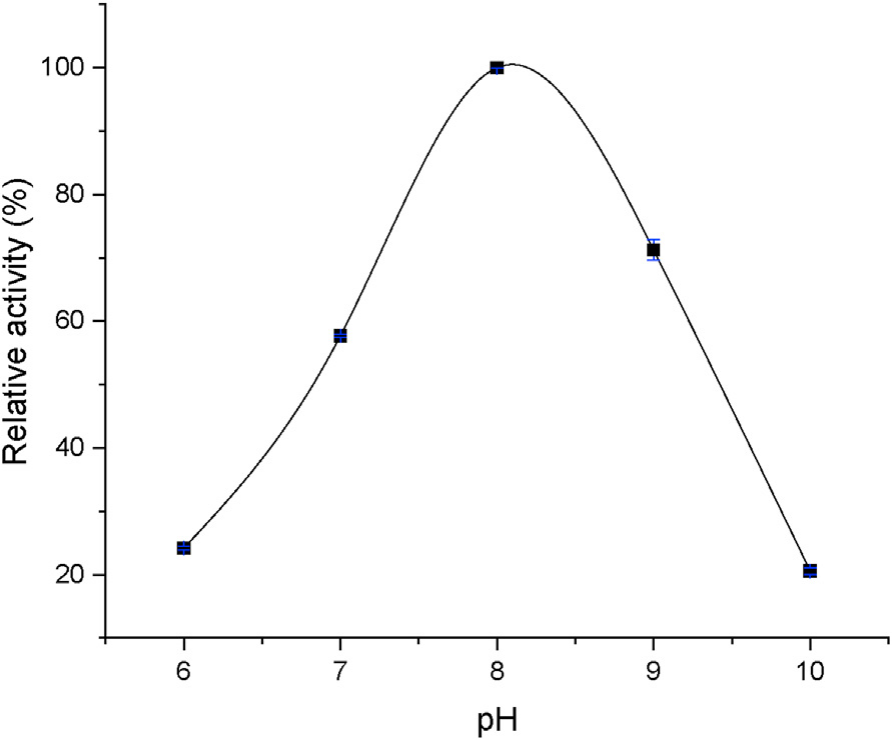
Relative esterase activity of the enzyme on various pH. The assay was performed at 80 °C using pNP-decanoate as substrate.

**Figure 10:**
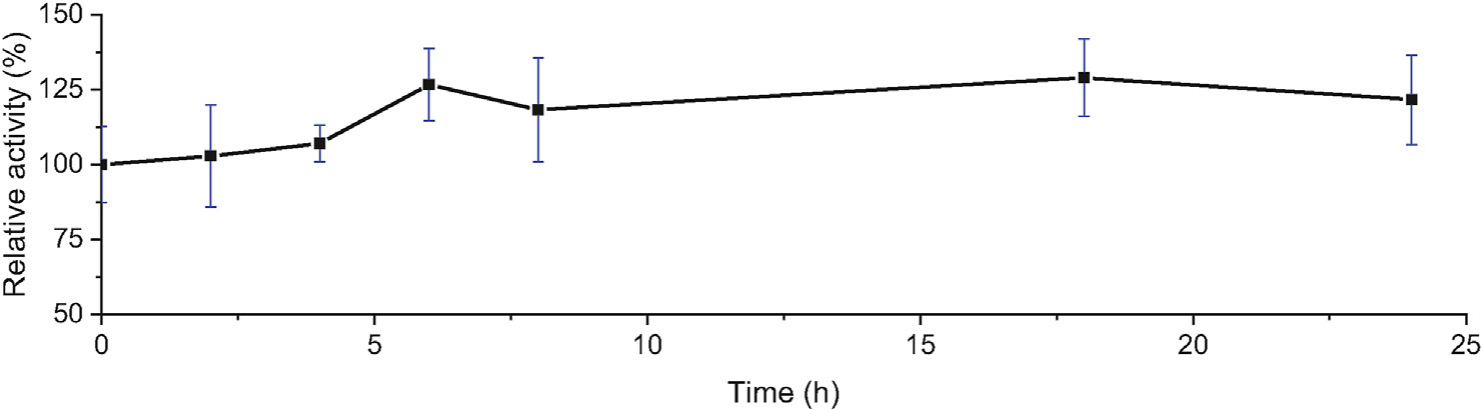
Relative esterase activity of the enzyme after periode incubation at 80 °C. The assay was performed using pNP-decanoate as substrate.

Further characterization on the activities of TesITB showed that the enzyme was extremely thermostable and alkaline tolerant with optimum temperature at 80 °C (Figure 8) and pH 8 (Figure 9). Further characterization on thermostability by incubating of the enzyme at 80 °C showed that the activity was relatively stable up to 24 h (Figure 10). At first 6 h of incubation appeared that the activity showed an elevation by increasing time incubation. Thermostability of the enzyme is understandable since total genome used to clone the gene was isolated from hot spring at 92–95 °C [24]. However, the enzyme showing an alkaline tolerant was surprising since the hot spring is acidic environment with pH 2–4. This suggesting that naturally the enzyme was expressed as an intracellular enzyme. Thermostability and alkaline tolerant are usually influenced by flexibility of molecular conformation [38]. The fact that TesITB showed an extreme thermostable probably due to the structure of the enzyme is very rigid. This is supported by molecular modelling showing that the enzyme was more stable on nonpolar solvent. Most of thioesterase reported are non-thermostable enzyme [8, 10, 39]. Based on *in silico* analysis, TesITB showed low homology with other thioesterase (Table 1) but contain most of residues required as thioesterase group. It might probably the comformation of TesITB is unlikely similar with other thioesterases. An esterolytic enzyme, namely Est DZ3, was reported [40] to show high thermostability with optimum activity at 75 °C and pH 8, however the enzyme prefer C4 substrate.

**Figure 11:**
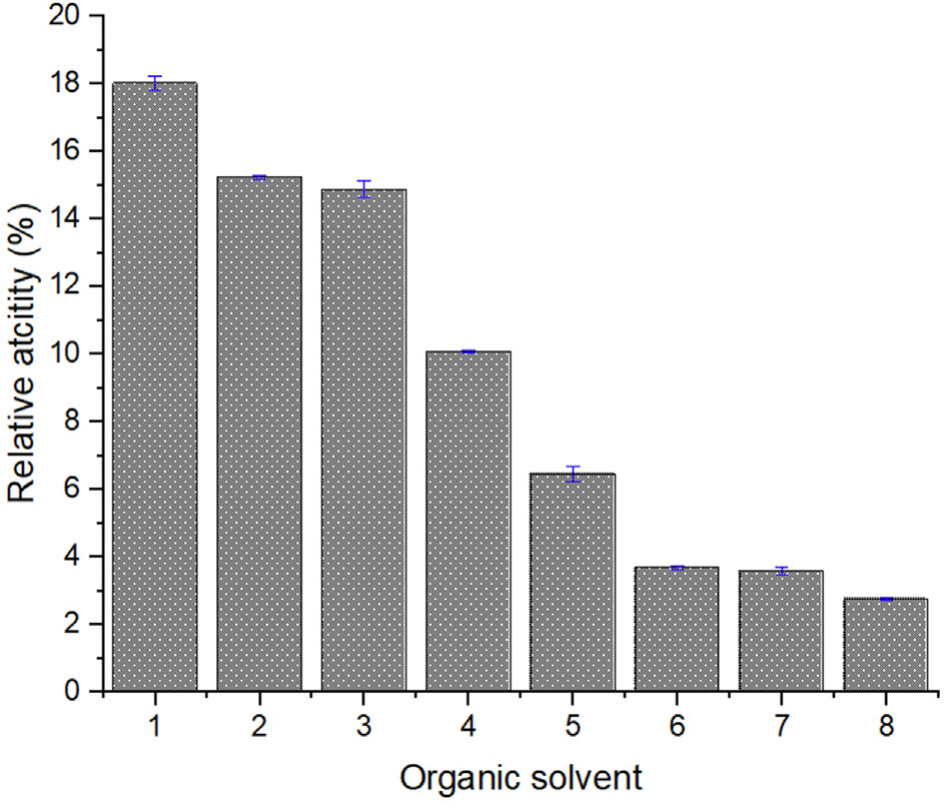
Relative esterase activity of the enzyme on the present of various organic solvent. (1) Methanol (2) Ethanol (3) n-buthanol (4) Isopropanol (5) Acetonitrile (6) Acetone (7) Chloroform (8) n-hexane. The assay was performed at pH 8, 80 °C using pNP-decanoat as substrate.

**Figure 12:**
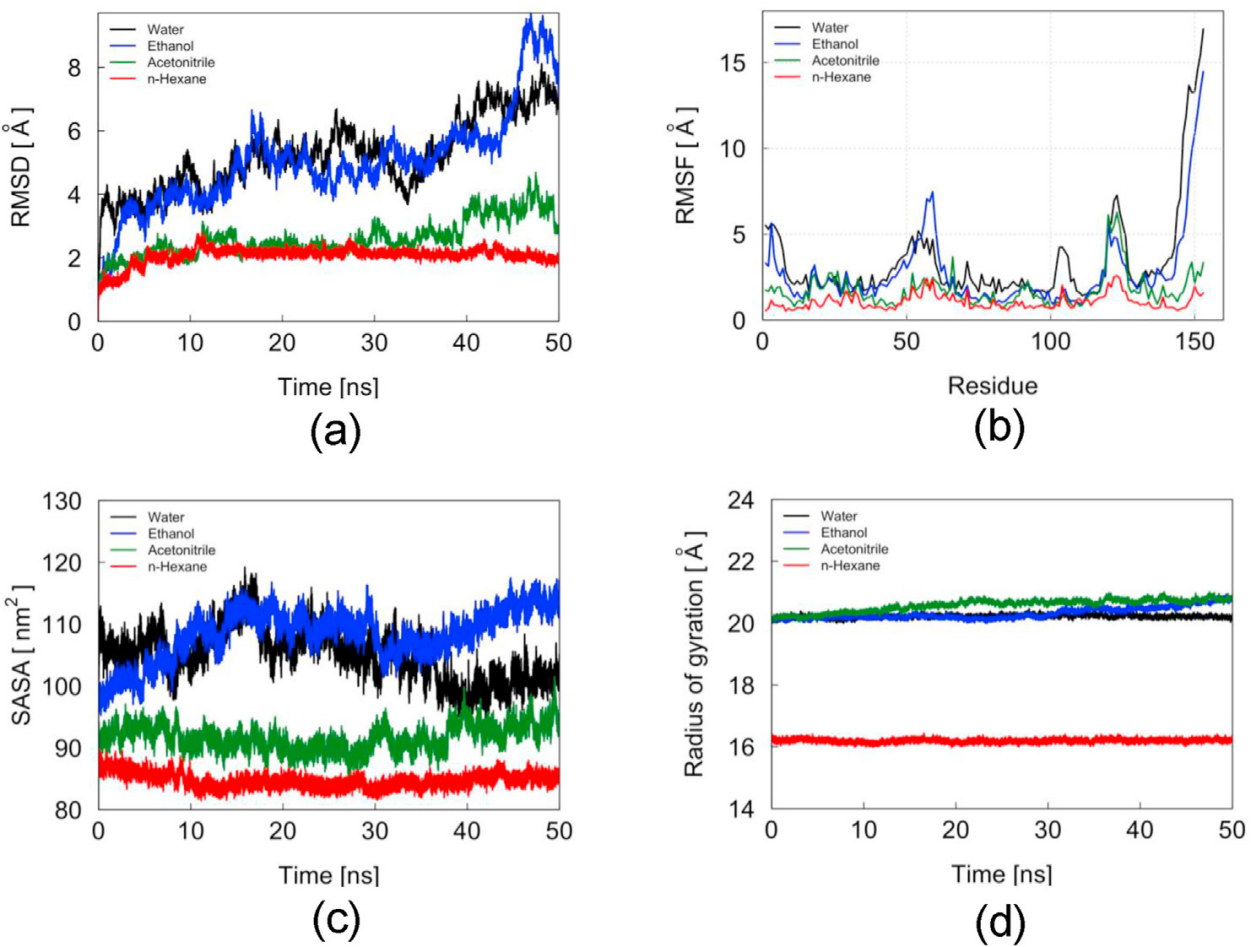
Molecular Dynamic simulations of TesITB on various organic solvents (a) Root mean square deviations of TesITB dissolved in water, ethanol, acetonitrile, and *n*-hexane. (b) Root mean square fluctuations of TesITB dissolved in water, ethanol, acetonitrile, and *n*-hexane. (c) Solvent accessible surface area of TesITB for all regions. (d) The radius of gyration of TesITB protein.

Most of enzyme are inactivated in the presence of organic solvent, however, some ester hydrolysis still remain functional since the enzymes have rigid conformation [41]. TesITB exhibited activity in polar organic solvent (Figure 11). The activity was decreased by reducing polarity of the solvent. In the present of chloroform or n-hexana the activity remained around 15% compared to that in methanol. To further probe stability on organic solvent, molecular dynamic simulation was measured on TesITB structure using four adopted solvents (water, ethanol, acetonitrile and n-hexane). The result showed that TesITB appeared highly dynamic in water and ethanol at 353 K based on the RMSD value (Figure 12a). At 353 K, flexibility of amino acid residues of TesITB in water and ethanol are higher compared to that in acetonitrile and n-hexane (Figure 12b) based on values of root mean square fluctuation. Solvent accessible surface area (SASA) is the surface area of the protein that might be reached by the solvent moieties. The SASA values of TesITB in water and ethanol are larger than in acetonitrile and n-hexane (Figure 12c). The evidence is in agreement with RMSF values showing that the flexibility of amino acid residues of TesITB in n-hexane is lower than in other solvents. Furthermore, radius gyration of TesITB is approximately 20 A° in water solvent (Figure 12d). The aforementioned value is not significantly different compared to that in ethanol and acetonitrile. Radius gyration is a quantity showing the compactness of a 3-dimensional structure. The radius gyration of TesITB is significantly smaller when the protein is dissolved in n-hexane. It indicates that the protein structure is shrinking in n-hexane. In general, it is suggested that TesITB structure is able to adapt itself in various organic solvent. The protein has a significant dynamic in ethanol compared to other adopted solvents. Therefore, it is presumed that a high activity of TesITB is highly supported by the presence of polar organic solvents. Ester hydrolase from *Pseudomonas sp* still showed high activity in the present of methanol up to 30% concentration [14], meanwhile ester hydrolase from *Geobacillus thermoleovorans* showed inactive in the presence of n-hexane or chloroform [42]. Remaining 15% activity of TesITB in chloroform or n-hexane suggesting that conformation of TesITB is unflexible and rigid.

TesITB is highly thermostable and alkaline tolerant enzyme. The enzyme still showed an activity on n-hexane as solvent. This is suggested that the structure of the enzyme is rigid and inflexible. Homological analysis of TesITB with other knows thioesterase only showing 66% homolog however TesITB contains all residues required as a group of thioesterase. All of the data suggested that TesITB is a novel thioesterase.

## 4 Conclusion

A novel thioesterase gene from a natural sample (Domas hot spring) was cloned and expressed in *E. coli*. Homological analysis of the protein appeared close to thioesterase family with the highest similarity to thioesterase from uncultured *Acidilobus sp* (66%). Phylogenetic analysis of the sequence showed that the protein forms same group with *Acidilobus* thioesterase, however, detail analysis showed that the protein was out of branch with other known thioesterase. The protein exhibited thioesterase and ester hydrolysis activities. The optimum activity of the enzyme was at 80 °C, pH 8. The activity of the enzyme remained stable after incubation at 80 °C, up to 24 h. In addition, the activity of the enzyme remained high in the present of polar organic solvent.

## Declarations

### Author contribution statement

Suharti Suharti: Conceived and designed the experiments; Performed the experiments; Analyzed and interpreted the data; Wrote the paper.

Gita Mahardika: Performed the experiments; Analyzed and interpreted the data; Wrote the paper.

Heni Yohandini: Performed the experiments.

Made Puspasari Widhiastuty: Analyzed and interpreted the data; Contributed reagents, materials, analysis tools or data.

Laksmi Dewi, Raissa Raissa: Performed the experiments; Analyzed and interpreted the data.

Akhmaloka Akhmaloka: Conceived and designed the experiments; Performed the experiments; Analyzed and interpreted the data; Contributed reagents, materials, analysis tools or data; Wrote the paper.

Raden Aditya Wibawa Sakti, Setyanto Tri Wahyudi: Analyzed and interpreted the data.

### Funding statement

This work was supported by P3MI Research Grant, 10.13039/501100015689Institut Teknologi Bandung, 10.13039/501100009509Ministry of Research, Technology and Higher Education, Republic of Indonesia. Gita Mahardika was supported by 10.13039/501100009509Ministry of Research, Technology and Higher Education, Republic of Indonesia.

### Data availability statement

Data included in article/supplementary material/referenced in article.

### Declaration of interests statement

The authors declare no conflict of interest.

### Additional information

No additional information is available for this paper.
